# ﻿Three new species of the genus *Perinereis* (Annelida, Nereididae) from Egyptian coasts

**DOI:** 10.3897/zookeys.1132.87629

**Published:** 2022-11-29

**Authors:** Asmaa Haris Elgetany, Torsten H. Struck, Christopher J. Glasby

**Affiliations:** 1 Zoology Department, Faculty of Science, Damietta University, New Damietta, Central zone, 34517, Egypt Damietta University New Damietta Egypt; 2 Natural History Museum, University of Oslo, P.O. Box 1172, Blindern, 0318 Oslo, Norway University of Oslo Oslo Norway; 3 Museum and Art Gallery of the Northern Territory, PO Box 4646, Darwin NT 0800, Australia Museum and Art Gallery of the Northern Territory Darwin Australia; 4 Currently, Australia Museum, 1 William Street, Darlinghurst NSW 2010, Australia Australia Museum Darlinghurst Australia

**Keywords:** Integrative taxonomy, morphometrics, *Perinereisdamietta* sp. nov., *Perinereisfayedensis* sp. nov., *Perinereisnuntia* species complex, *Perinereissuezensis* sp. nov., systematics

## Abstract

Despite being one of the most common groups of polychaetes on intertidal shores, the genus *Perinereis* (Nereididae) is comparatively poorly known taxonomically, with confusion still existing due to the lack of comprehensive systematic studies. The systematics of *Perinereis* species from the intertidal Egyptian coasts of the Red Sea, Gulf of Suez and Suez Canal have been investigated using morphology and the mitochondrial barcoding marker cytochrome oxidase subunit I (COI). New sequence data was obtained for 102 *Perinereis* specimens and analysis included all publicly available COI data from other *Perinereis* species. The COI data indicate that monophyly of the *P.nuntia* species group is doubtful, as specimens identified in this species group from south-eastern Asia and Australia form a monophyletic group exclusive of the three new species described in this study from the Red Sea region. A morphometric character set (26 characters) was used to identify and characterize each specimen in the study. Three distinct morphospecies belonging to the *P.nuntia* species group were found, each differentiated by the number and type of paragnaths on pharyngeal areas V and VI, relative sizes of parapodial lobes, type of notochaetae and neurochaetae, and form of the neurochaetal falciger blades. The three morphospecies were well supported by COI data: two of the three new species, *Perinereissuezensis***sp. nov.** and *Perinereisfayedensis***sp. nov.**, are closely similar to *P.nuntia* sensu stricto, while the other, *Perinereisdamietta***sp. nov.**, is similar to *P.heterodonta*. The new species are described and illustrated, and bring the number of species in *Perinereis* to 97. The new species are compared and contrasted to the closely similar *P.heterodonta*, *P.nuntia* and other congeners from the region.

## ﻿Introduction

The family Nereididae includes several highly variable species characterized by high intra-specific morphological variation especially associated with the pharynx (e.g., number and arrangement of paragnaths) or associated with the parapodia (relative proportions of lobes/ligules and form of chaetae) and coloration. Often these morphologically variable species also show differences in reproductive biology (Yoshida 1984; Hardege and Bartels-Hardege 1995) and have widespread distributions. Such species were recognized in the old taxonomic literature as ‘forms’ or ‘varieties’ of a species (e.g., Fauvel 1932), but today most are recognized as full species (Read and Fauchald 2022). More than other nereidid genera, *Perinereis* contains a number of ‘species-groups’ or ‘species complexes’ (e.g. Wilson 1993; Glasby et al. 2013), which have served to group like forms, although none have been shown to be monophyletic.

*Perinereis* Kinberg, 1865 is the second most species-rich genus in the family. It includes approximately 94 worldwide-distributed valid species (Villalobos-Guerrero 2019, Villalobos-Guerrero et al. 2021; Bakken et al. 2022; Conde-Vela 2022). Bakken and Wilson (2005) found that the genus was likely to be polyphyletic based on morphology; specifically, that *P.nuntia* Lamarck, 1818 together with *P.variodentata* Augener, 1913, and *P.vallata* Grube, 1857 were more closely related to *Neanthes/Nereis* species than to the type species of the genus. Subsequent molecular studies have either supported non-monophyly (Glasby et al. 2013), or refuted it (Alves et al. 2020), but both studies lacked in-depth taxon sampling as they did not specifically set out to test *Perinereis* monophyly. Tosuji et al. (2019) found support for a clade among western Pacific members of the *P.nuntia* species group, suggesting biogeographic support for splitting the species group, although as *P.nuntia* s. s. was not included in the analysis, the question of whether the species group is monophyletic remained open.

Members of *Perinereis* have been long recognized based primarily on the number and type of paragnaths on areas V and VI (e.g., Kinberg 1865; Grube 1878; Horst 1889). Specifically, *Perinereis* have well-separated, mostly conical paragnaths on both pharyngeal rings and bar-shaped (which are shield-shaped) paragnaths on area VI (Villalobos-Guerrero et al. 2021). Other important characters are the number of paragnath bands on area VII-VIII, the presence of merged paragnaths on area IV, the presence of isolated paragnaths on area III, type and relative sizes of parapodial lobes (particularly dorsal ligule), type and form of the neurochaetal spinigers and falcigers, and presence of teeth on the jaws (Hutchings et al. 1991; Bakken and Wilson 2005; Santos et al. 2005; Sampértegui et al. 2013). Those species having more than two bars (often many more) on area VI have been considered traditionally as varieties of *P.nuntia* (Savigny in Lamarck, 1818) (e.g., Fauvel 1919, 1921, 1932, 1953; Augener 1931). Nowadays, they are all recognized as species belonging to the *Perinereisnuntia* species complex (Wilson 1993; Wilson and Glasby 1993; Glasby and Hsieh 2006; Villalobos-Guerrero 2019).

Until recently, the *Perinereisnuntia* species group comprised 15 valid species (Wilson and Glasby 1993; Glasby and Hsieh 2006; Tosuji et al. 2019). It is characterized by the presence of an arc of bar-shaped (including shield-shaped) paragnaths (or a mixture of bars/shields and cones) on area VI (Tosuji et al. 2019). Subsequent revision involving a broader re-examination of the *P.nuntia* species group has revealed a further two members, historically referred to under *Neanthes* Kinberg, 1865 [Nereis (Nereis) latipalpa Schmarda, 1861 from Cape Town, South Africa, and Nereis (Neanthes) larentukana Grube in Peters, 1881 from Larantuka, Flores, Indonesia (Villalobos-Guerrero 2019)]. In total, Villalobos-Guerrero recognized five new combinations, bringing to 20 the number of valid species in the *P.nuntia* species group.

The present study investigates the taxonomy of three putative species belonging to the *P.nuntia* species group sampled from the Gulf of Suez, Suez Canal, and the northern Red Sea using a detailed morphological study and the mitochondrial barcoding marker cytochrome oxidase subunit I (COI). We compare our material with other members of the species group originally described from the region, including *Perinereisnuntia* (type locality: Gulf of Suez) and *P.heterodonta* Gravier, 1899 (type locality: Red Sea, Obock, Gulf of Aden, Djibouti,). Our results show that all three species are new to science: two of them, *P.suezensis* sp. nov. and *P.fayedensis* sp. nov. from Gulf of Suez (part of Red Sea), are closely similar to *P.nuntia*, while the other one, *P.damietta* sp. nov., from Hurghada (northern Red Sea), is more similar to *P.heterodonta*. The three species are described below.

## ﻿Material and methods

### ﻿Data collection and preservation

**Figure 1.**
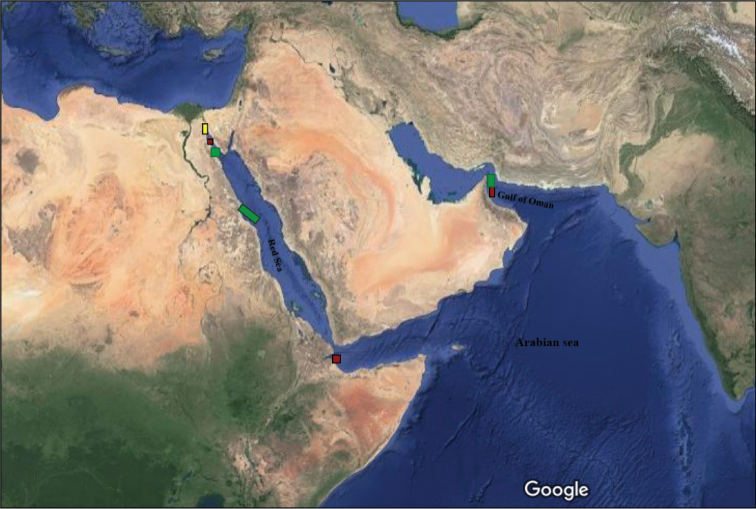
Map of *Perinereis* species localities referred to in this study. Colored squares indicate different species: red – *Perinereisheterodonta*, green – *P.nuntia*, yellow – *P.damietta* sp. nov., *P.suezensis* sp. nov. and *P.fayedensis* sp. nov. (see Table 1). Map based on URL: https://www.google.com.eg/maps/@18.940384,68.1599381,10852298m/data=!3m1!1e3?hl=en.

Sampling was carried out during the period of January 2015 to July 2017 from four localities along the intertidal zone of Egyptian coasts of the Red Sea, Gulf of Suez and Suez Canal (Fig. 1).

A section of the mid body was removed from the Red Sea specimens and stored in 96% ethanol for molecular analysis. The rest of the animal was fixed in 4% seawater formalin and stored in 70% ethanol for morphological studies.

### ﻿Morphological examination

Specimens were studied and photographed using a stereo microscope, Leica MZ16, with a Planapo 1.0X and Canon EOS 500D, as well as a compound microscope, Leica DFC420 connected to a Leica Computer CTR600 and a DM600B camera. For morphological characterization, we grouped specimens into three putative species, and recorded 26 morphometric characters for a subset of 45 of the 102 specimens in total (Suppl. material 1, Table 1). Measurements included number of chaetigers, total body length (cm), jaw length (mm), width at chaetiger 10 excluding parapodia (cm), number of paragnaths on area I, area II (left), area II (right), area III, area IV (left), area IV (right), area IV, area V, area VI (left), area VI (right), and area VII–VIII, length of dorsal cirri (DC; along its ventral edge from the proximal junction with the DNL to the distal extremity) at chaetiger 10 (mm), length of dorsal notopodial ligule (DNL; along its dorsal edge from the proximal junction with the DC to the distal extremity, as per Villalobos-Guerrero (2019: fig. 4e)) at chaetiger 10 (mm), ratio DC/DNL, length of dorsal cirri of one chaetiger between chaetigers 75–90 (mm), length of dorsal notopodial ligule of one chaetiger between chaetigers 75–90 (mm), ratio DC/DNL, length of postero-dorsal tentacular cirrus (as indicated by chaetiger reached when posteriorly extended), occurrence of subacicular heterogomph spiniger at chaetiger 10, occurrence of subacicular heterogomph spiniger at chaetigers 75–90, chaetiger of first occurrence of subacicular heterogomph spiniger. We also recorded the form of the notopodial glands in posterior parapodia, and the color pattern in preserved species. Observation of features on the non-everted pharynx required a longitudinal dissection in the mid-ventral oral region. Characters reported in the taxonomic descriptions are those of the holotype, with those of the paratypes in parentheses. Terminology for nereidid features followed Hylleberg et al. (1986), Bakken and Wilson (2005), Bakken et al. (2009), Villalobos-Guerrero and Bakken (2018), and Villalobos-Guerrero (2019).

**Table 1.** Species used in this study with Sample ID for the new specimens and accession numbers for all specimens.

Genus	Species	Sample ID	Accession #	Genus	Species	Accession #
*Perinereis*	*damietta* sp. nov.	5-1	OP610122	*Perinereis*	*aibuhitensis*	KC800611
*Perinereis*	*damietta* sp. nov.	5-2	OP610123	*Perinereis*	*aibuhitensis*	KC800612
*Perinereis*	*damietta* sp. nov.	5-8	OP610124	*Perinereis*	*aibuhitensis*	KC800613
*Perinereis*	*damietta* sp. nov.	5-10	OP610125	*Perinereis*	*aibuhitensis*	KC800614
*Perinereis*	*damietta* sp. nov.	12-1B	OP610126	*Perinereis*	*aibuhitensis*	KC840698
*Perinereis*	*damietta* sp. nov.	12-2B	OP610127	*Perinereis*	*aibuhitensis*	KF611806
*Perinereis*	*damietta* sp. nov.	12-3B	OP610128	*Perinereis*	*aibuhitensis*	KY129885
*Perinereis*	*damietta* sp. nov.	12-4B	OP610129	*Perinereis*	*aibuhitensis*	MN256534
*Perinereis*	*damietta* sp. nov.	12-5B	OP610130	*Perinereis*	*aibuhitensis*	MN256535
*Perinereis*	*damietta* sp. nov.	12-6B	OP610131	*Perinereis*	*aibuhitensis*	MN256536
*Perinereis*	*damietta* sp. nov.	12-8B	OP610132	*Perinereis*	*aibuhitensis*	MT511716
*Perinereis*	*damietta* sp. nov.	12-10B	OP610133	*Perinereis*	*aibuhitensis*	MT511717
*Perinereis*	*damietta* sp. nov.	16-1	OP610134	*Perinereis*	*aibuhitensis*	MT511718
*Perinereis*	*damietta* sp. nov.	16-2	OP610135	*Perinereis*	*aibuhitensis*	MT712474
*Perinereis*	*damietta* sp. nov.	16-3	OP610136	*Perinereis*	*aibuhitensis*	MW593148
*Perinereis*	*damietta* sp. nov.	16-4	OP610137	*Perinereis*	*anderssoni*	MH143495
*Perinereis*	*damietta* sp. nov.	R4-1	OP610138	*Perinereis*	*anderssoni*	MH143497
*Perinereis*	*damietta* sp. nov.	R4-2	OP610139	*Perinereis*	*anderssoni*	MH143498
*Perinereis*	*damietta* sp. nov.	R4-3	OP610140	*Perinereis*	*anderssoni*	MH143502
*Perinereis*	*damietta* sp. nov.	R6-1	OP610141	*Perinereis*	*anderssoni*	MH143503
*Perinereis*	*damietta* sp. nov.	R6-2	OP610142	*Perinereis*	*anderssoni*	MH143504
*Perinereis*	*damietta* sp. nov.	R6-3	OP610143	*Perinereis*	*anderssoni*	MH143507
*Perinereis*	*damietta* sp. nov.	R8-1	OP610144	*Perinereis*	*anderssoni*	MH143508
*Perinereis*	*damietta* sp. nov.	R8-2	OP610145	*Perinereis*	*anderssoni*	MH143514
*Perinereis*	*damietta* sp. nov.	R8-3	OP610146	*Perinereis*	*anderssoni*	MH143516
*Perinereis*	*damietta* sp. nov.	R8-5	OP610147	*Perinereis*	*anderssoni*	MH143520
*Perinereis*	*damietta* sp. nov.	R8-6	OP610148	*Perinereis*	*anderssoni*	MH143522
*Perinereis*	*damietta* sp. nov.	R8-7	OP610149	*Perinereis*	*brevicirris*	JX503024
*Perinereis*	*damietta* sp. nov.	R8-8	OP610150	*Perinereis*	*brevicirris*	JX966314
*Perinereis*	*suezensis* sp. nov.	1-9	OP612948	*Perinereis*	*brevicirris*	KC800628
*Perinereis*	*suezensis* sp. nov.	2-3	OP612949	*Perinereis*	*brevicirris*	KC800630
*Perinereis*	*suezensis* sp. nov.	2-4	OP612950	*Perinereis*	*brevicirris*	KC800632
*Perinereis*	*suezensis* sp. nov.	2-5	OP612951	*Perinereis*	*camiguinoides*	KF850496
*Perinereis*	*suezensis* sp. nov.	2-6	OP612952	*Perinereis*	*cultrifera*	KC800624
*Perinereis*	*suezensis* sp. nov.	2-7	OP612953	*Perinereis*	*cultrifera*	KC800625
*Perinereis*	*suezensis* sp. nov.	2-8	OP612954	*Perinereis*	*cultrifera*	KC800627
*Perinereis*	*suezensis* sp. nov.	5-4	OP612955	*Perinereis*	*cultrifera*	KR916906
*Perinereis*	*suezensis* sp. nov.	5-5	OP612956	*Perinereis*	*cultrifera*	KR916907
*Perinereis*	*suezensis* sp. nov.	5-6	OP612957	*Perinereis*	*cultrifera*	KR916908
*Perinereis*	*suezensis* sp. nov.	5-7	OP612958	*Perinereis*	*cultrifera*	KR916909
*Perinereis*	*suezensis* sp. nov.	5-9	OP612959	*Perinereis*	*cultrifera*	KR916910
*Perinereis*	*suezensis* sp. nov.	7-5B	OP612960	*Perinereis*	*cultrifera*	KR916911
*Perinereis*	*suezensis* sp. nov.	7-7B	OP612961	*Perinereis*	*cultrifera*	KR916912
*Perinereis*	*suezensis* sp. nov.	7-8B	OP612962	*Perinereis*	*cultrifera*	KY129882
*Perinereis*	*suezensis* sp. nov.	7-9B	OP612963	*Perinereis*	*cultrifera*	KY129883
*Perinereis*	*suezensis* sp. nov.	7-10B	OP612964	*Perinereis*	*cultrifera*	MN256544
*Perinereis*	*suezensis* sp. nov.	8-1	OP612965	*Perinereis*	*cultrifera*	MN256545
*Perinereis*	*suezensis* sp. nov.	8-2	OP612966	*Perinereis*	*cultrifera*	NC_051994
*Perinereis*	*suezensis* sp. nov.	8-3	OP612967	*Perinereis*	*curvata*	MW277905
*Perinereis*	*suezensis* sp. nov.	8-4	OP612968	*Perinereis*	*euiini*	KY249122
*Perinereis*	*suezensis* sp. nov.	8-5	OP612969	*Perinereis*	*euiini*	KY249123
*Perinereis*	*suezensis* sp. nov.	8-6	OP612970	*Perinereis*	*euiini*	KY249124
*Perinereis*	*suezensis* sp. nov.	8-7	OP612971	*Perinereis*	*falklandica*	HQ705184
*Perinereis*	*suezensis* sp. nov.	8-8	OP612972	*Perinereis*	*falklandica*	HQ705185
*Perinereis*	*suezensis* sp. nov.	8-9	OP612973	*Perinereis*	*gualpensis*	HQ705186
*Perinereis*	*suezensis* sp. nov.	8-10	OP612974	*Perinereis*	*gualpensis*	HQ705187
*Perinereis*	*suezensis* sp. nov.	10-3B	OP612975	*Perinereis*	*gualpensis*	HQ705188
*Perinereis*	*suezensis* sp. nov.	10-4B	OP612976	*Perinereis*	*helleri*	JX420256
*Perinereis*	*suezensis* sp. nov.	10-5B	OP612977	*Perinereis*	*linea*	MT511711
*Perinereis*	*suezensis* sp. nov.	10-6B	OP612978	*Perinereis*	linea	MT511712
*Perinereis*	*suezensis* sp. nov.	10-7B	OP612979	*Perinereis*	linea	MT511713
*Perinereis*	*suezensis* sp. nov.	10-8B	OP612980	*Perinereis*	linea	MT511714
*Perinereis*	*suezensis* sp. nov.	10-10B	OP612981	*Perinereis*	linea	MT511715
*Perinereis*	*suezensis* sp. nov.	11-1B	OP612982	*Perinereis*	*longidonta*	HQ705190
*Perinereis*	*suezensis* sp. nov.	11-3B	OP612983	*Perinereis*	longidonta	HQ705191
*Perinereis*	*suezensis* sp. nov.	11-4B	OP612984	*Perinereis*	*nuntia*	JX420257
*Perinereis*	*suezensis* sp. nov.	11-5B	OP612985	*Perinereis*	*nuntia*	JX644015
*Perinereis*	*suezensis* sp. nov.	12-9B	OP612986	*Perinereis*	*nuntia*	MH337359
*Perinereis*	*suezensis* sp. nov.	14-1	OP612987	*Perinereis*	*seridentata*	JF293314
*Perinereis*	*suezensis* sp. nov.	14-2	OP612988	*Perinereis*	*singaporiensis*	EU835665
*Perinereis*	*suezensis* sp. nov.	14-3	OP612989	*Perinereis*	sp.	EU352319
*Perinereis*	*suezensis* sp. nov.	14-4	OP612990	*Perinereis*	sp.	KR916903
*Perinereis*	*suezensis* sp. nov.	14-5	OP612991	*Perinereis*	sp.	KR916904
*Perinereis*	*suezensis* sp. nov.	14-6	OP612992	*Perinereis*	sp.	KR916905
*Perinereis*	*suezensis* sp. nov.	14-7	OP612993	*Perinereis*	sp.	KX525487
*Perinereis*	*suezensis* sp. nov.	14-8	OP612994	*Perinereis*	sp.	KX525497
*Perinereis*	*suezensis* sp. nov.	14-9	OP612995	*Perinereis*	sp.	KX525498
*Perinereis*	*suezensis* sp. nov.	14-10	OP612996	*Perinereis*	sp.	KX525499
*Perinereis*	*suezensis* sp. nov.	18-1	OP612997	*Perinereis*	sp.	KX840014
*Perinereis*	*suezensis* sp. nov.	18-2	OP612998	*Perinereis*	sp.	MH143496
*Perinereis*	*suezensis* sp. nov.	18-3	OP612999	*Perinereis*	sp.	MH143499
*Perinereis*	*suezensis* sp. nov.	18-4	OP613000	*Perinereis*	sp.	MH143500
*Perinereis*	*suezensis* sp. nov.	18-5	OP613001	*Perinereis*	sp.	MH143501
*Perinereis*	*suezensis* sp. nov.	18-6	OP613002	*Perinereis*	sp.	MH143505
*Perinereis*	*suezensis* sp. nov.	18-7	OP613003	*Perinereis*	sp.	MH143506
*Perinereis*	*suezensis* sp. nov.	18-8	OP613004	*Perinereis*	sp.	MH143509
*Perinereis*	*suezensis* sp. nov.	18-9	OP613005	*Perinereis*	sp.	MH143510
*Perinereis*	*suezensis* sp. nov.	18-10	OP613006	*Perinereis*	sp.	MH143511
*Perinereis*	*suezensis* sp. nov.	R5-1	OP613007	*Perinereis*	sp.	MH143512
*Perinereis*	*suezensis* sp. nov.	R5-2	OP613008	*Perinereis*	sp.	MH143513
*Perinereis*	*suezensis* sp. nov.	R5-3	OP613009	*Perinereis*	sp.	MH143515
*Perinereis*	*suezensis* sp. nov.	R5-4	OP613010	*Perinereis*	sp.	MH143517
*Perinereis*	*suezensis* sp. nov.	R6-4	OP613011	*Perinereis*	sp.	MH143518
*Perinereis*	*fayedensis* sp. nov.	2-2	OP605755	*Perinereis*	sp.	MH143519
*Perinereis*	*fayedensis* sp. nov.	5-3	OP605756	*Perinereis*	sp.	MH143521
*Perinereis*	*fayedensis* sp. nov.	7-4B	OP605757	*Perinereis*	sp.	MH143523
*Perinereis*	*fayedensis* sp. nov.	7-6B	OP605758	*Perinereis*	sp.	MH143524
*Perinereis*	*fayedensis* sp. nov.	10-1B	OP605759	*Perinereis*	sp.	MH143525
*Perinereis*	*fayedensis* sp. nov.	10-2B	OP605760	*Perinereis*	sp.	MH143526
*Perinereis*	*fayedensis* sp. nov.	10-9B	OP605761	*Perinereis*	sp.	MN823962
*Perinereis*	*fayedensis* sp. nov.	11-2B	OP605762	*Perinereis*	sp.	MT528267
*Perinereis*	*fayedensis* sp. nov.	12-7B	OP605763	*Perinereis*	sp.	OK430976
*Perinereis*	*aibuhitensis*		GU362686	*Perinereis*	*suluana*	JX392072
*Perinereis*	*aibuhitensis*		JX503021	*Perinereis*	*suluana*	JX420245
*Perinereis*	*aibuhitensis*		JX503022	*Perinereis*	*suluana*	JX420246
*Perinereis*	*aibuhitensis*		JX503023	*Perinereis*	*suluana*	JX420247
*Perinereis*	*aibuhitensis*		JX661442	*Perinereis*	*suluana*	JX420248
*Perinereis*	*aibuhitensis*		JX661443	*Perinereis*	*suluana*	JX420250
*Perinereis*	*aibuhitensis*		JX661444	*Perinereis*	*suluana*	JX420251
*Perinereis*	*aibuhitensis*		JX661445	*Perinereis*	*suluana*	JX420252
*Perinereis*	*aibuhitensis*		JX661446	*Perinereis*	*suluana*	JX420253
*Perinereis*	*aibuhitensis*		JX661447	*Perinereis*	*suluana*	JX420254
*Perinereis*	*aibuhitensis*		JX661448	*Perinereis*	*suluana*	JX420255
*Perinereis*	*aibuhitensis*		JX661449	*Perinereis*	*vallata*	HQ705192
*Perinereis*	*aibuhitensis*		JX661450	*Perinereis*	*vallata*	HQ705196
*Perinereis*	*aibuhitensis*		JX661451	*Perinereis*	*vallata*	JX676119
*Perinereis*	*aibuhitensis*		JX661452	*Perinereis*	*vallata*	JX676143
*Perinereis*	*aibuhitensis*		JX661453	*Perinereis*	*vallata*	MT511721
*Perinereis*	*aibuhitensis*		JX661454	*Perinereis*	*vallata*	MT511722
*Perinereis*	*aibuhitensis*		JX661455	*Perinereis*	*vancaurica*	MT511719
*Perinereis*	*aibuhitensis*		JX661456	*Perinereis*	*wilsoni*	KC800623
*Perinereis*	*aibuhitensis*		JX661457	*Perinereis*	*wilsoni*	KC800629
*Perinereis*	*aibuhitensis*		JX661458	*Perinereis*	*wilsoni*	KC800631
*Perinereis*	*aibuhitensis*		JX661459	*Perinereis*	*wilsoni*	KY129887
*Perinereis*	*aibuhitensis*		JX661460	*Perinereis*	*wilsoni*	KY129888
*Perinereis*	*aibuhitensis*		JX661461	*Perinereis*	*wilsoni*	KY129889
*Perinereis*	*aibuhitensis*		JX661462	*Perinereis*	*wilsoni*	MN256541
*Perinereis*	*aibuhitensis*		JX661463	*Perinereis*	*wilsoni*	MN256542
*Perinereis*	*aibuhitensis*		JX661464	*Perinereis*	*wilsoni*	MN256543
*Perinereis*	*aibuhitensis*		JX661465	*Dendronereis*	*chipolini*	MW5320841
*Perinereis*	*aibuhitensis*		JX661466	*Hediste*	*japonica*	MN876864
*Perinereis*	*aibuhitensis*		JX661467	*Namalycastis*	*abiuma*	KU351089
*Perinereis*	*aibuhitensis*		JX661468	*Platynereis*	*dumerilii*	AF178678
*Perinereis*	*aibuhitensis*		JX661469			

### ﻿Institutional abbreviations

Samples are deposited in the Damietta University - Faculty of Science (**DUFS**), Damietta, Egypt, and the Senckenberg Forschungsinstitut und Naturmuseum (**SMZ**), Frankfurt, Germany.

### ﻿Molecular study

Genomic DNA was extracted from three to four segments of the middle section of each worm using the DNeasy Tissue Kit (Qiagen) according to manufacturers’ instructions with at least two elution steps to increase the amount of DNA. For each individual, the nucleotide sequences of the mitochondrial COI were amplified using the primer pair LCO1490JJ (forward, 5’-CHA CWA AYC ATA AAG ATA RYG G-3’) and HCO2198JJ (reverse, 5’-AWA CTT CVG GRT GVC CAA ARA ATC A-3’) (Astrin and Stuben 2008). The PCR was carried out in a reaction volume of a 20 µl solution each with 3.8 µl water, 2 µl Q solution, 10 µl Qiagen Multiplex-Solution, 1.6 µl 10 pmol/µl LCO1490JJ, 1.6 µl 10 pmol/µl HCO2198JJ and 1µl template DNA. PCR parameters were 95 °C for 15min, 15 cycles of (94 °C for 35s, 55 °C for 90s with “-1 °C decrease per cycle”, 72 °C for 90s), 25 cycle of (94 °C for 35s, 50 °C for 90s, 72 °C for 90s) and 72 °C for 10min. The PCR product was purified using ExoProStar (Qiagen, Hilden, Germany). Both strands were sequenced using Sanger sequencing at Macrogen Inc. (South Korea). Sequences were assembled into contigs using CodonCode Aligner v. 6.0.2 (Centerville, MA). The 102 new COI sequences were deposited at National Center for Biotechnology Information (NCBI) (Table 1).

For the phylogenetic analyses, we included all publicly available COI data from other specimens of *Perinereis* as well as five nereidid species, who have a complete mitochondrial genome sequenced, as outgroup taxon (Table 1). The sequences were aligned using the multiple sequence alignment software MAFFT v. 7.310 (Katoh and Standley 2013) with an automatic selection of the best alignment method and the option ‘globalpair’. The selected alignment strategy was FFT-NS-i plus an iterative refinement method of two cycles. The 5' and 3' prime ends of the resulting aligned, where trimmed until the first position at each having at least 90% consensus (i.e., < 10% of taxa with missing data at the ends). The final dataset had 267 sequences and 583 nucleotide positions. A maximum likelihood (**ML**) analysis was conducted with IQ-TREE v. 1.6.12 using the automatically selecting the best-fitting substitution model and an ultrafast bootstrap analysis with 1000 pseudoreplicates (Nguyen et al. 2015; Kalyaanamoorthy et al. 2017; Thi Hoang et al. 2018). The selected model was GTR+F+I+G4 (GTR substitution model with ML estimated frequencies, a proportion of invariant sites and a Gamma distribution with four categories).

## ﻿Results

### ﻿Phylogenetic analysis

**Figure 2.**
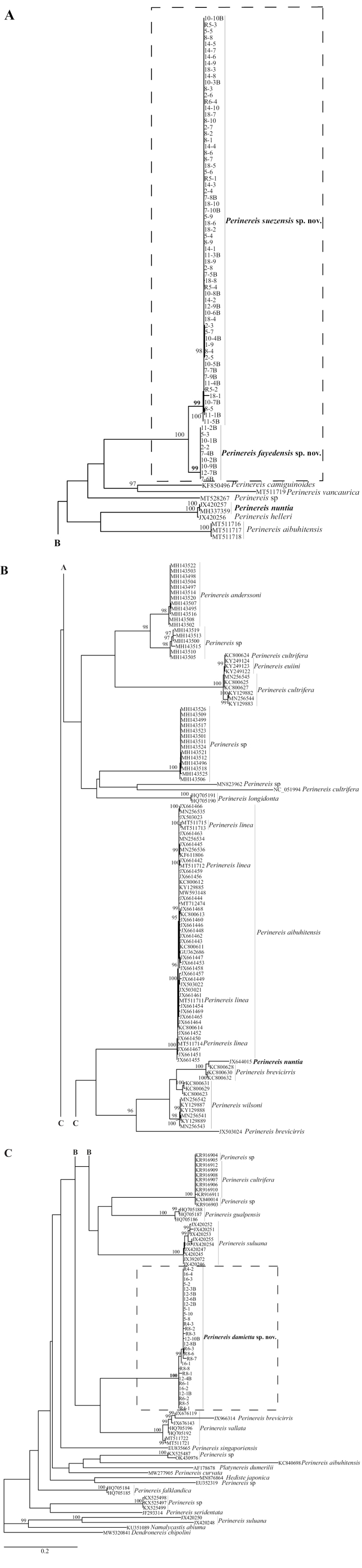
ML tree (logL = -11297.8710) of the *Perinereis* species in this analysis shown in three parts (**A, B, C**). The points, where we cut the branches, are indicated by letters. The new species *P.suezensis* sp. nov., *P.fayedensis* sp. nov., and *P.damietta* sp. nov. are highlighted by boxes and in bold. *Perinerisnuntia* is also highlighted in bold. Bootstrap values equal to and more than 95 indicating strong support are given at the branches. The three relevant bootstrap values are highlighted in bold. The scale bar shows substitutions/position.

The ML tree (logL = -11297.8710) showed that the genus *Perinereis* is probably not monophyletic as the outgroup *Hedistejaponica* Izuka, 1908 grouped within the genus (Fig. 2), but bootstrap support is low for most basal nodes with values below 95. While the monophyly of several *Perinereis* species is strongly supported with values equal to or more than 95, the monophyly of the *P.nuntia* species group and of several other species seem to be doubtful or, alternatively, specimens have been wrongly assigned to species. For example, the specimen JX644015 of *P.nuntia* is placed within *P.brevicirris* (Grube, 1866) (see Remarks for *Perinereissuezensis* sp. nov.). Other cases comprise species, for example, of *P.aibuhitensis* (Grube, 1878), *P.suluana* (Horst,1924), *P.brevicirris*, *P.wilsoni* (Glasby & Hsieh, 2006), *P.cultrifera* (Grube, 1840), or *P.euiini* (Park & Kim, 2017). Hence, the requirement for a thorough taxonomic revision of the genus is further supported. The specimens collected for this study were grouped into three strongly supported clades, which were supported with bootstrap values of 99, 99 and 100, respectively (boxes in Fig. 2A, C). Herein, we describe them as new species, *P.suezensis* sp. nov., *P.fayedensis* sp. nov. and *P.damietta* sp. nov. Moreover, *P.suezensis* sp. nov. and *P.fayedensis* sp. nov. are sister groups to each other with a maximal bootstrap of 100.

### ﻿Taxonomic account

**Nereididae Blainville, 1818**

**Nereidinae Blainville, 1818**

***Perinereis*** K**inberg, 1856**

#### ﻿ Perinereis nuntia

Taxon classificationAnimaliaPhyllodocidaNereididae

species complex

1F0099B5-C23D-5138-B935-90F785127E50

Perinereis nuntia species complex Wilson & Glasby, 1993: 259. – Glasby and Hsieh 2006: 558. – Villalobos-Guerrero 2019: 468.

##### Diagnosis.

*Perinereis* species having conical paragnaths on all areas (rarely absent on area V), except area VI with long bars, which can be shield-shaped or pyramidal paragnaths, arranged in a single-arched row; area V with paragnaths displaced posteriorly (on everted proboscis) to those on area VI; area IV rarely with merged paragnaths.

##### Remarks.

We have restricted the diagnosis of the species complex to include only unique diagnostic features. Some new characters introduced by Villalobos-Guerrero (2019) describing the faint ridges and furrows of the dorsal oral ring may prove to be useful when broader comparisons can be made. However, at this stage we consider that the form of the pharyngeal ridges and furrows is too closely allied to underlying musculature, and therefore could be unduly influenced by the fixation process and length of time in preservative. Similarly, the form (and length) of the deeply embedded paired nuchal organs may prove to be useful when more comparative data are available. However, observation of that character depends heavily on state of preservation (e.g., they are seen more clearly in specimens relaxed before preservation); in the present specimens the nuchal organs were hidden under the anterior edge of the apodous segment and thus not visible externally. Pharyngeal morphologies are reported herein by describing the form and arrangement of paragnaths on the ridges and in the furrows of the pharynx. The form and arrangement of paragnaths on area VI is unique to the genus (and family) and serves as the easiest way to recognize a member of the species complex. However, Tosuji et al. (2019) have demonstrated that in at least two East Asian species of the complex, the number of bars increases with the growth of individuals (fragmentation of the long bars produces multiple shorter bars (= shield-shaped paragnaths)). Therefore, this character should be used cautiously for species identification across the group, and comparisons are best made between individuals of similar size until we have a better understanding of the processes involved.

#### ﻿ Perinereis suezensis sp. nov.

Taxon classificationAnimaliaPhyllodocidaNereididae

CE79C976-CEB8-572A-BC4B-F756693D7039

https://zoobank.org/E765642E-72D6-41FD-AB9D-C9607F3E48CB

Fig. 3

##### Material examined.

***Holotype***: DUFS 067 Al-Adabiya; west of Port Taofik, Gulf of Suez (Red Sea), intertidal, under coarse sands, at 29°56'06.0"N, 32°28'36.6"E, collection date (15.01.2015) ***Paratypes***: 13 specimens (DUFS 057-066, 068-070) from Al-Qantara, Suez Canal, intertidal, muddy sand bottom, at 30°50'31.5"N, 32°18'54.8"E, Fayed, western shore of Great Bitter Lake, intertidal, silty mud bottom at 30°20'18.0"N, 32°18'14.9"E, and Al-Adabiya (same collection details as holotype). Collection dates (18.02.2015/ 15.01.2015/ 01.07.2017).

##### Non-type material.

2 specimens (SMZ unregistered), Hurghada, Egypt (northern Red Sea), at 27°15'42.0"N, 33°48'44.7"E, intertidal, under stones, St. 9a, ‘3192', det. as *Nereis* sp., collected 9.01.1992

##### Description.

***Holotype*** (DUFS 067) not complete, 53 chaetigers, 50 mm in length, 2 mm wide at chaetiger 10 ***Paratypes*** with 33–88 chaetigers, 32–81 mm long, 2.0–4.5 mm wide at chaetiger 10. Epidermis with orange pigmentation on anterior dorsum in some preserved paratypes.

Prostomium with entire anterior margin; as wide as long. Antennae closely set, as long as ~ 1/3 length of prostomium. Eyes black, anterior pair set slightly further apart than posterior pair; lenses not obvious.

Apodous segment ~ 1.2× or 1.6× longer than chaetiger 1. Posterodorsal tentacular cirri extending back to chaetiger 6 (6–7).

**Figure 3.**
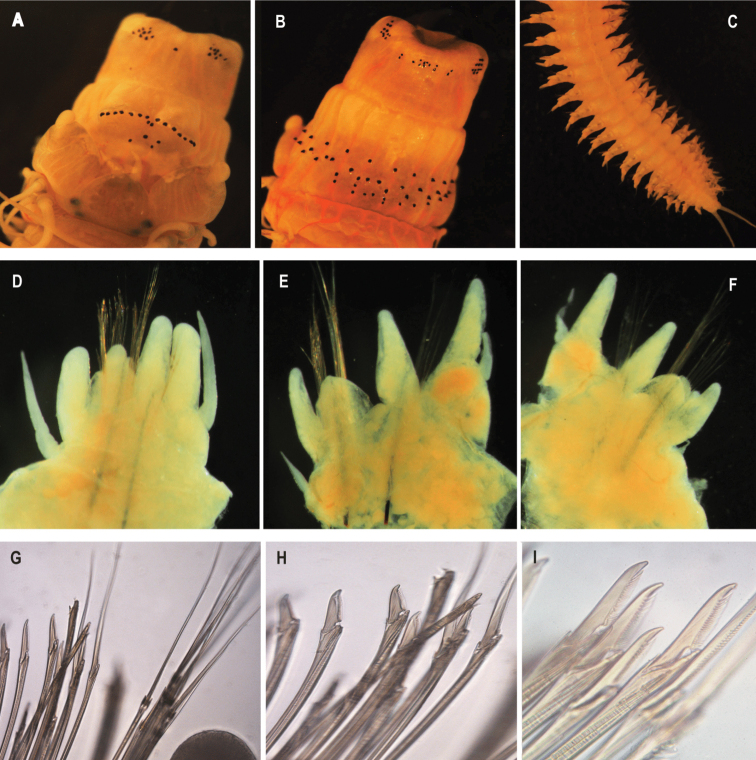
*Perinereissuezensis* sp. nov. All pictures are from the holotype if not stated otherwise **A** anterior end, maxillary apparatus, dorsal view **B** anterior end, maxillary apparatus, ventral view **C** posterior end, ventral view **D** right parapodium, posterior view, chaetiger 7 **E** right parapodium, anterior view, chaetiger 33 **F** right parapodium, anterior view, chaetiger 64 **G** chaetal bundle of a right parapodium homogomph spiniger, heterogomph spiniger & heterogomph falciger, chaetiger 7 **H** neuropodial sub-acicular heterogomph falciger, chaetiger 7 **I** heterogomph falciger, chaetiger 7.

Pharynx with jaws translucent, red-brown, with 7 (7–8) teeth. Paragnaths black. Area I with 2 (1–5) conical paragnaths; area II conical paragnaths with 5 (5–10) on left and 8 (7–10) on right, in a triangular patch; area III with 10 (9–17) conical paragnaths in 2–3 rows, with two laterally isolated paragnaths; area IV conical paragnaths with 16 (11–19) on left, 13 (9–16) on right, in two or three rows, in elongated triangle; area V with 4 (2–4) conical paragnaths interspersed with one or two bars, set well proximal (on everted proboscis) to line of area VI paragnaths; area VI with 15 (14–21), shield-shaped bars with pointed tips (very close in appearance to cones), arranged in one arc, with the right and left rows almost touching; area VII-VIII with 44 (37–44) conical paragnaths arranged in a single band of two rows laterally to three or four rows deep medially (Fig. 3A, B; Table 2). Paragnath-free region between areas VI and VII-VIII broad, ca. as wide as palpophore; paragnaths of VII-VIII not visible in dorsal view (Fig. 3A).

**Table 2.** Comparison of key characters between forms resembling *P.heterodonta* (pale grey) and *Perinereisnuntia* (dark grey) in the Red Sea, the Gulf of Aden, and the Arabian (= Persian) Gulf. Abbreviations: AIII = Area III; AV = Area V; AVI Area VI; AVII-VIII = Areas VII and VIII; p-dTC = posterior extension of posterodorsal tentacular cirri (chaetiger); ratio of lengths of dorsal cirri v dorsal notopodial lobe in posterior chaetigers; HS, presence (p) or absence (a) of heterogomph spinigers in anterior neuropodia; NA = data not available.

Species	Type locality	AIII (lateral group p/a)	AV	AVI	AVII-VIII	p-dTC	p-DC:DNL	HS	Reference
*heterodonta* sensu stricto	Djibouti	6-7 irregular cluster	0	10–16	18	5	NA	a	Gravier (1899)
*heterodonta* sensu stricto	Gulf of Oman	8-14 (p) cluster	0–1	14–24	20–35	1–6	0.6–1×	a	Yousefi et al. (2011); CJG pers. obs. 2021
*Perinereisdamietta* sp. nov.	Gulf of Suez	3-9 (one transverse row)	0–1	24–40	16–32	2–4	1.1–1.2	a	Present paper
*djiboutiensis*	Djibouti	small rectangular patch (p)	0	6–7	NA (3 rows)	10–15	NA (DC > DNL)	NA	Fauvel (1919)
*nuntia* sensu stricto	Gulf of Suez, Red Sea	15	3(2–4)	10–12 (8–10)	41 (36–50)	(4–6)	4–5X (3–4X)	p	Savigny in Lamarck (1818); Villalobos-Guerrero (2019) [in parentheses]
*nuntia* sensu Wilson and Glasby 1993	‘Red Sea’	8-14 (p)	3–4	8–13	24–31	6–14	~1.2	p	Wilson and Glasby (1993)
*nuntia* sensu Yousefi et al. 2011	Gulf of Oman	9-15	3	13–20	36–58	8–14	2X	p	Yousefi et al. (2011); CJG pers. obs. 2021
*Perinereissuezensis* sp. nov.	Gulf of Suez, Red Sea	9-17 (p)	2–4	14–21	37–44	6–7	1.0–1.3X	p	Present paper
*Perinereisfayedensis* sp. nov.	Gulf of Suez, Red Sea	2-5 (one tranverse row)	1–4	14–17	28–40	6–8	1.1–1.2X	p	Present paper

Anterior notopodia with conical dorsal and median ligules of equal length in anterior body; dorsal ligule slightly longer in mid- and posterior body. Superior lobes absent. DC length 1.1 (1.0–1.2) × DNL length anteriorly (chaetigers 10–20); posteriorly DC length 1.09 (1.0–1.3) × length of DNL length (chaetigers 75–90). DC and DNL of mid-body parapodia proportionally similar to those of posterior parapodia (Fig. 3D–F).

Dorsal notopodial ligule larger than ventral notopodial ligule anteriorly and posteriorly. Neuropodia with inferior and postchaetal lobes, ventral ligule and ventral cirri. Neuropodial postchaetal lobe lowly rounded, not projecting beyond end of acicular ligule. Ventral neuropodial ligule subconical, ca. as long as median ligule throughout. Ventral cirri extending laterally to reach tip of ventral neuropodial ligule anteriorly, extending to ~ 1/4 length of ventral neuropodial ligule posteriorly (Fig. 3D–F).

Notochaetae with homogomph spinigers throughout, blades long; teeth short. Neurochaetae in upper fascicle with homogomph spinigers with long blades; one heterogomph falciger with short blades throughout, blades serrated. Neurochaetae in lower fascicle with heterogomph falcigers, blades short and thick, teeth long; and two or three heterogomph spinigers, median long blades, teeth short present throughout body. Aciculae black, single in each ramus (Fig. 3G–I).

Pygidium with anal cirri extending to last 6 (6–7) chaetigers, 5 (5–7) mm long, whitish cream without any pigmentation (Fig. 3C).

##### Variation (non-type material).

Two specimens: one complete with 105 chaetigers, 57 mm long and 2.8 mm wide, and another with regenerating tail, 107 chaetigers, 71 mm long and 4.3 mm wide. Apodous segment ~ 1.3–1.8× longer than chaetiger 1. Posterodorsal tentacular cirri extending back to chaetigers 5 and 6. Jaws with 4–7 teeth. Paragnaths count: area I with 2; area II with 8–17 on left and 9–17 on right; area III with 11 or 12 in two or three rows; area IV with 17 or 18 on both sides, in two or three rows; area V with three or four; area VI with 8–12 on left and 8–11 on right, shield-shaped bars with pointed tips and cones arranged in one row with the right and left side rows almost touching each other; area VII-VIII with 47 or 48, arranged in a single band of two rows laterally to three or four rows deep medially. Dorsal cirrus length ~ 0.8× length of dorsal notopodial ligule anteriorly and 0.7–0.9× length of dorsal notopodial ligule posteriorly. Ventral cirri extending laterally to reach tip or half-length of ventral neuropodial ligule anteriorly. Neurochaetae in upper fascicle with 1–3 heterogomph falcigers. Neurochaetae in lower fascicle with 1–4 heterogomph spinigers, rarely absent.

##### Distribution and habitat.

Gulf of Suez, Suez Canal including Great Bitter Lake, northern Red Sea; intertidal sand and mud, under stones.

##### Etymology.

The new species is named after the port city of Suez (Egyptian Arabic pronunciation: (السويس) located on the north coast of the Gulf of Suez.

##### Remarks.

The molecular data place *P.suezensis* sp. nov. clearly apart from all other species and the monophyly of the species is very well supported by a bootstrap value of 99 (Fig. 2A). Not considering identical sequences between specimens within each species, the average genetic distance based on the branch length in the tree to its sister-taxon, *P.fayedensis* sp. nov., is 6.65% (± 0.60%), while the average genetic distance within *P.suezensis* is only 0.24% (± 0.37%). Hence, there is a clear gap in the genetic distances.

In addition to our sequences, only three additional COI sequences for *P.nuntia* have been published: JX420257 (Indonesia), JX644015 (South Korea), and MH337359 (Andaman and Nicobar Islands). JX420257 and MH337359 are identical (bootstrap value of 100; Fig. 2A), however, they are distantly related to *P.suezensis* sp. nov. (Fig. 2A). Glasby et al. (2013) found that *P.nuntia*JX420257 clustered with *P.helleri* (Grube, 1878), and together was the sister group of *P.suluana*, both relationships with a high Bayesian posterior probability (> 0.95). This confirms the distant relationship between material identified as *P.nuntia* from the Australasian region. JX644015 nested within a group comprising otherwise only *P.brevicirris* with a bootstrap value of 100 (Fig. 2B). Together they clustered with the East Asian-restricted *P.wilsoni*. Hence, it is also dubious whether JX644015 is a *P.nuntia* specimen and perhaps should be considered to belong to a species related to other East Asian *Perinereis* based on the molecular data. Reports of *P.brevicirris*, which was considered a synonym of *P.vallata* by Wilson and Glasby (1993) but is now accepted as valid (see key in Villalobos-Guerrero 2019), are widespread throughout the Indo-Pacific but most tropical and northern hemisphere records are unlikely to represent this species, which was originally described from Ile Saint Paul, Southern Ocean.

The new species is most similar to *P.nuntia*, which was also described from the Gulf of Suez. Although the exact location of Savigny’s specimens has never been established, it is very likely to be from shallow waters of the port city of Suez, as for Savigny’s other polychaetes (see Villalobos-Guerrero 2019 and references therein). *Perinereisnuntia* was recently redescribed by Villalobos-Guerrero (2019), and based on his redescription and Lamarck’s type description, we have found two key differences between the two species (values in parentheses those of Villalobos-Guerrero). The number and shape of paragnaths in area VI: 14–21shield-shaped paragnaths in the new species, compared to 8–10 (10–12) short bars in *P.nuntia*; and the relative length of the posterior dorsal cirri, which are 1.0–1.3× the DNL in the new species and 4–5 (3–4) × the DNL in *P.nuntia*. The new species also shows similarities with *P.heterodonta* from Djibouti in having a high number of paragnaths on area VI and short dorsal cirri in the posterior end; however, the new species can be differentiated from *P.heterodonta* by the greater number of paragnaths on areas V (24 vs. 0-1) and VII-VIII (37–44 vs. 18–35) (Table 2).

The larger-sized, non-type specimens generally had more paragnaths in each area compared to the type material, except for area VI. The fewer paragnaths in area VI in the non-type specimens is most likely due to loss, as the ones present were irregularly spaced, with some gaps large enough to accommodate a lost shield-shaped bar or two cones. Another reflection on the condition of the non-type specimens is the unusually short dorsal and ventral cirri; on this point, the cirri appeared withered and many were missing, which we attribute to damage or a fixation artifact.

#### ﻿ Perinereis fayedensis sp. nov.

Taxon classificationAnimaliaPhyllodocidaNereididae

42D8A37B-13B1-5611-B77C-BB66C6677320

https://zoobank.org/92062163-4B3D-46B3-9D2D-9DC669BD73F5

Fig. 4

##### Material examined.

***Holotype***: DUFS 0123 Al-Adabiya, west of Port Taofik, Gulf of Suez (Red Sea), intertidal, under coarse sands, at 29°56'06.0"N, 32°28'36.6"E. ***Paratypes*** (DUFS 120–122, 124–128): 8 specimens from El-Qantara, Suez Canal, intertidal, muddy sand bottom, at 30°50'31.5"N, 32°18'54.8"E, Fayed, western shore of Great Bitter Lake, intertidal, silty mud bottom, at 30°20'18.0"N, 32°18'14.9"E, Al-Adabiya (same collection details as holotype).

##### Description.

***Holotype*** (DUFS 0123) not complete, 49 chaetigers, 35 mm in length, 3 mm wide at chaetiger 10. ***Paratypes*** with 37–88 chaetigers, 30–70 mm long, 1.5–4.5 mm wide at chaetiger 10. Epidermis whitish cream with a longitudinal beige pigmentation stripe on ventral side of posterior chaetigers in some preserved.

Prostomium with entire anterior margin; wide as long. Antennae closely set, as long as ~ 1/3 length of prostomium. Eyes black, anterior pair set slightly further apart than posterior pair; lenses not obvious.

Apodous segment ~ 1.5× longer than chaetiger 1. Posterodorsal tentacular cirri with distinct cirrophores, extend back to chaetiger 7 (6–8).

**Figure 4.**
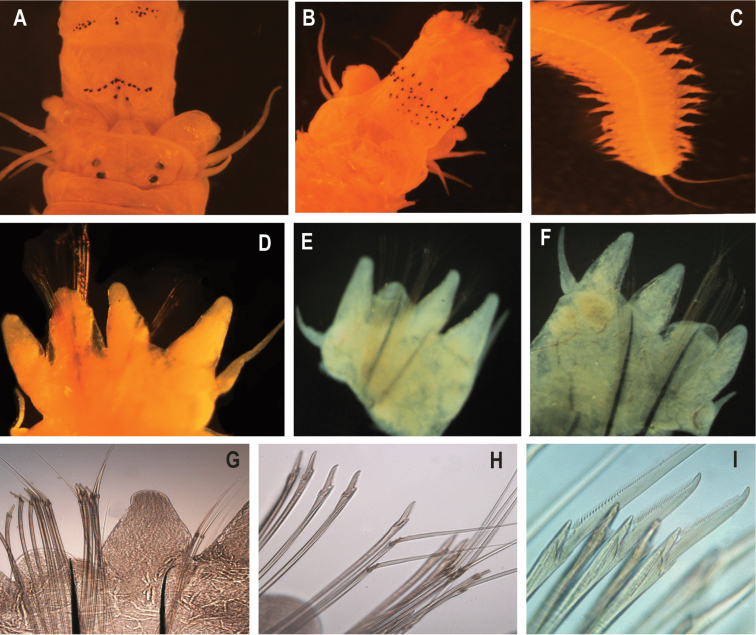
*Perinereisfayedensis* sp. nov. All pictures are from the holotype if not stated otherwise **A** anterior end, maxillary apparatus, dorsal view **B** anterior end, maxillary apparatus, ventral view **C** posterior end, ventral view **D** right parapodium, anterior view, chaetiger 16 **E** right parapodium, posterior view, chaetiger 32 **F** right parapodium, posterior view, chaetiger 67 **G** chaetal bundle of a right parapodium, homogomph spiniger and heterogomph falcigers, chaetiger 17 **H** neuropodial chaetal bundle of a right parapodium, homogomph spiniger & heterogomph falciger, chaetiger 33 **I** heterogomph falciger, chaetiger 33.

Pharynx with jaws translucent red-brown, with 8 (7–8) teeth. Maxillary ring of pharynx with paragnaths, arranged in discrete areas, areas II-IV arranged in regular comb-like rows. Area I with 2 (1–2) conical paragnaths in vertical arrangement; area II with 9 (7–10) in left and 9 (7–10) in right conical paragnaths, three or four rows in a triangular patch; area III with 2 (2–5) conical paragnaths in vertical arrangement; area IV with 13 (12–15) in left, 14 (12–15) in right, conical paragnaths without bars; area V with 4 (1–3) conical paragnaths; area VI with 17 (14–17), shield-shaped bars with pointed tip present, cones paragnaths absent; area VII-VIII with 38 (28–40) conical paragnaths with small p-bars interspersed arranged in a single band of 3–5 rows (Fig. 4A, B; Table 2). Paragnath-free region between areas VI and VII-VIII broad, ca. as wide as palpophore; paragnaths of VII-VIII not visible in dorsal view (Fig. 4A).

Notopodia with conical dorsal and median ligules of equal length throughout. Superior lobes absent. DC length 1.2 (0.9–1.2) × length of DNL length anteriorly (chaetigers 10–20); posteriorly, DC length 1.2 (1.1–1.2) × length of DNL length (chaetigers 75–90). DC and DNL of mid-body parapodia proportionally similar to those of posterior parapodia (Fig. 4D–F).

Neuropodia with inferior and postchaetal lobes, ventral ligule and ventral cirri. Neuropodial postchaetal lobe lowly rounded, not projecting beyond end of acicular ligule. Ventral neuropodial ligule subconical, ca. as long as median ligule throughout. Ventral cirri extending laterally to halfway to tip of ventral neuropodial ligule in anterior and midbody, extending to ~ 1/3 length of ventral neuropodial ligule posteriorly (Fig. 4D–F).

Aciculae black, single in each ramus (Fig. 4G). Notochaeta with homogomph spinigers throughout, spinigers of long blades; teeth short. Neurochaetae with homogomph spinigers and heterogomph falcigers in the supra and sub-acicular fascicle (Fig. 4G–I). Acicula black, single in each ramus.

Pygidium with anal cirri fine, tapering, extending to last 7 (6–8) chaetigers, 50 (45–55) mm long (Fig. 4C).

##### Remarks.

The molecular data place the new species, *P.fayedensis*, clearly apart from all other species and as sister to *P.suezensis* (Fig. 2A). The monophyly is well supported by a bootstrap value of 99. Not considering identical sequences between specimens within each species, the average genetic distance to its sister-taxon, *P.suezensis*, is 6.65% (± 0.60%), while the average genetic distance within *P.fayedensis* is substantially lower with a value of 0.01% (± 0.00%). Hence, there is again a clear gap in the genetic distances.

Morphologically, *P.fayedensis* is intermediate between *P.nuntia* and *P.heterodonta* described from Obock, Djibouti, Gulf of Aden. It differs from the former most notably in the number of paragnaths in area III (2–5 vs. ~ 15 in *P.nuntia*) and area VI (14–17 vs. 8–12 in *P.nuntia*), and the relative length of the DC (1.1–1.2× DNL in the new species vs. 3–5× DNL in *P.nuntia*; Table 2). *Perinereisfayedensis* can be distinguished from *P.heterodonta* by having fewer paragnaths in area III (2–5 in one row vs. a cluster of 6–7 in *P.heterodonta*) and more paragnaths in area VII-VIII (28–40 vs. 18 in *P.heterodonta*) (see Table 2).

##### Distribution and habitat.

Gulf of Suez, Suez Canal including Great Bitter Lake; intertidal sand and mud, under stones.

##### Etymology.

The new species is named after the Egyptian city of Fayed on the western shore of Great Bitter Lake approximately halfway along the Suez Canal.

#### ﻿ Perinereis damietta sp. nov.

Taxon classificationAnimaliaPhyllodocidaNereididae

84ED1C9B-2EFB-5DDC-B96F-F09B7AA30E3A

https://zoobank.org/32CCF83E-CDF3-4A3E-802A-E5C1EDD8B851

Fig. 5

##### Material examined.

***Holotype***: DUFS 055, Hurghada (northern Red Sea), Grand Aquarium beach, subtidal area, clay bottom, at 27°07'59.2"N, 33°49'51.2"E. ***Paratypes***: 22 specimens (DUFS 027–048) and non-type material 6 specimens (DUFS 049-054) from Al-Adabiya, west of Port Taofik, Gulf of Suez (Red Sea), intertidal, under coarse sands, at 29°56'06.0"N, 32°28'36.6"E and from Hurghada, National institute of Oceanography beach, intertidal and upper subtidal area, from muddy and sand bottoms, at 27°17'03.1"N, 33°46'19.8"E (Egypt).

##### Description.

***Holotype*** (DUFS 055) not complete, 94 chaetigers, 62 mm in length, 4.5 mm wide at chaetiger 10. ***Paratypes*** with 42–96 chaetigers for 30–115 mm long and 1.5–7 mm wide at chaetiger 10. Epidermis with orange and gold pigmentation on anterior dorsum and ventrum in some preserved samples.

Prostomium with entire anterior margin; relatively large, longer than wide, two pairs of eyes, dark green with black lenses, and two large palps longer than antennae, palpostyles conical. Antennae closely set, as long as ~ 1/3 length of prostomium. Lenses not obvious.

One apodous anterior segment, ~ 1.6× longer than chaetiger 1. Tentacular cirri with distinct cirrophores, longest tentacular cirri extend back to chaetiger 2 (2–4).

**Figure 5.**
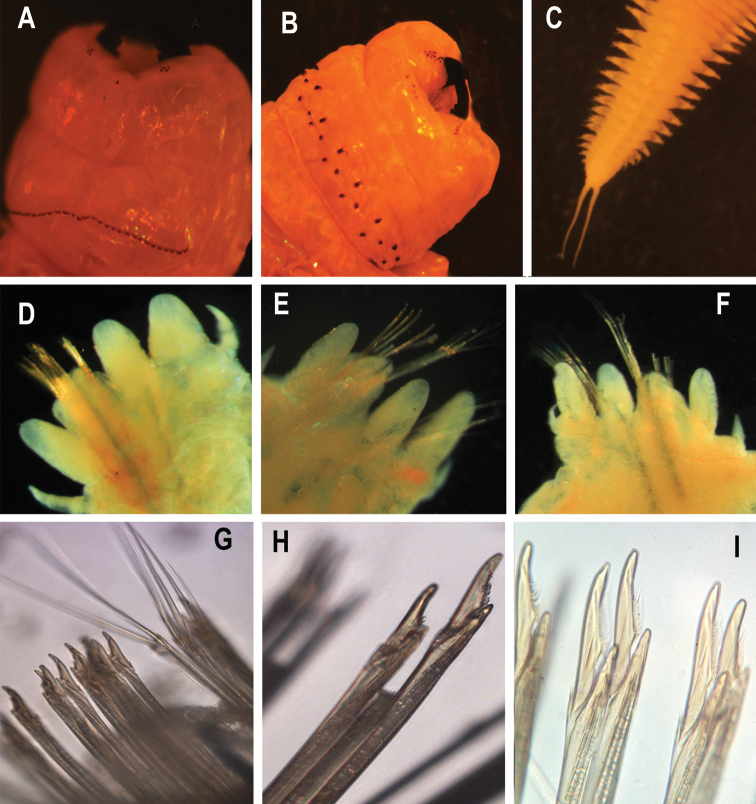
*Perinereisdamietta* sp. nov. All pictures are from the holotype if not stated otherwise **A** anterior end, maxillary apparatus, dorsal view **B** anterior end, maxillary apparatus, ventral view **C** posterior end, ventral view **D** right parapodium, posterior view, chaetiger 9 **E** right parapodium, posterior view, chaetiger 41 **F** right parapodium, anterior view, chaetiger 56 **G** chaetal bundle of a right parapodium, homogomph spiniger & heterogomph falciger chaetiger 56 **H** neuropodial chaetal bundle of a right parapodium, heterogomph falciger, chaetiger 56 **I** heterogomph falciger, chaetiger 67.

Pharynx with jaws black, 4 (4–5) reddish brown teeth. Paragnaths black with light brown base; those of maxillary ring pointed conical paragnaths. Paragnath counts: area I with 0 (0–2); area II with 2 (1–5) on the left side and 3 (2–5) on the right side; area III with 4 (3–9) in one transverse row; area IV with 14 (10–21) on the left side and 16 (10–20) on the right side; arranged in irregular row of unequal paragnaths. Area V with 0 (0–1); area VI with 24 (24–40) conical paragnaths arranged in one arc; area VII–VIII with 24 (16–32), similar in size, arranged in two rows (Fig. 5A, B). Paragnath-free region between areas VI and VII–VIII broad, ca. as wide as palpophore; paragnaths of VII–VIII not visible in dorsal view (Fig. 5A).

Anterior notopodia with conical dorsal and median ligules of equal length in anterior body; dorsal ligule slightly longer in mid- and posterior body. DC length 0.8 (0.7–1.0) × length of DNL length anteriorly (chaetigers 10–20); posteriorly, DC length 1.1 (0.9–1.2) × length of DNL (chaetigers 75–90). DC and DNL of mid-body parapodia proportionally similar to those of posterior parapodia (Fig. 5D).

Dorsal notopodial ligule; triangular with conical tip, slightly longer than notopodial ventral ligule throughout. Ventral notopodial ligule rounded triangular. Dorsal and ventral notopodial ligules marked decreasing in size on posterior chaetigers. Neuropodium with dorsal rounded lobe in anterior chaetigers, with one black acicula, less developed posteriorly. Ventral neuropodial ligule digitiform, similar in length to acicular ligule on anterior chaetigers; slightly longer than acicular ligule in posterior chaetigers. Ventral cirri extending to ~ 1/3 length of ventral neuropodial ligule anteriorly and posteriorly (Fig. 5D–F).

Notochaetae with homogomph spinigers, long and thin serrated blade throughout. Neurochaetae dorsal fascicle: homogomph spinigers; median thick serrated blade present and heterogomph falcigers present on anterior and posterior chaetigers, blades serrated. Neurochaetae ventral fascicle: heterogomph falcigers with median long and wide blades with a single terminal tooth, in anterior and posterior chaetigers (Fig. 5G–I). Aciculae black with red-brown base, single in each ramus.

Pygidium with anal cirri cirriform, cirri extending to last 2 (2–4) chaetigers (Fig. 5C).

##### Distribution and habitat.

Gulf of Suez, northern Red Sea; intertidal and subtidal, sand and mud, under stones.

##### Etymology.

The new species is named after the university of the first author, Damietta University, a noun in apposition. Damietta (Egyptian Arabic: *Dumyāț* (دمياط) is also a port city located on an eastern distributary of the Nile Delta, ~ 15 km from the Mediterranean Sea.

##### Remarks.

*Perinereisdamietta* sp. nov. is well supported by the highest bootstrap value of 100 (Fig. 2C) and clearly set apart from the other *Perinereis* species in the tree. According to the present molecular phylogeny, the sister group to *P.damietta* is *P.vallata*, which is also a former variety of *P.nuntia* (Wilson and Glasby 1993; Glasby and Hsieh 2006). Not considering identical sequences between specimens within each species, the average genetic distance to its sister group is 42.57% (± 6.72%), while the average genetic distance within *P.damietta* is 1.12% (± 0.74%) and hence substantially lower. Hence, there is again a clear gap in the genetic distances.

Herein, *P.vallata* also includes one specimen (JX966314) assigned to *P.brevicirris* (Fig. 2C). This is probably a misidentification given the very strong bootstrap support values of 100 for both the monophyly of *P.vallata* and the group of *P.brevicirris* specimens mentioned above (Fig. 2A, C).

*Perinereisdamietta* is morphologically most similar to *P.heterodonta* (type locality: Obock, Djibouti, Gulf of Aden). Both species belong to the group of the *P.nuntia* complex that lack heterogomph spinigers in anterior parapodia, which is unlike *P.nuntia*. Other key differences between *P.damietta* /*P.heterodonta* and *P.nuntia* are the shorter tentacular cirri and the fewer paragnaths in area V (0–1) (Table 2). *Perinereisdamietta* differs from *P.heterodonta* most notably in having 24–40 pyramidal paragnaths in area VI (vs. 10–16 in *P.heterodonta*). In this regard, it has the highest number of area VI paragnaths of any species in the *P.nuntia* species complex, exceeding the next highest (12–16 bars) found in *P.vallata* (Wilson and Glasby 1993).

Another species originally described from Djibouti, *Perinereisdjiboutiensis*, is unfortunately poorly known, especially in respect to the presence or absence of heterogomph spinigers in anterior parapodia and numbers of paragnaths in areas III and VII–VIII (Table 2). Although it resembles the new species in having one, or no, paragnaths in area V, it may be differentiated from the new species in having only six or seven short bars (may also include cone-shaped paragnaths) in area VI, which is the lowest of all species of the *Perinereisnuntia* species group in the region (Table 2), and in this regard it is closer to material described as *Perinereisnuntia* from the Red Sea by Wilson and Glasby (1993).

A novel character introduced by Villalobos-Guerrero (2019), the size of the gap between areas VI and VII–VIII, may also set this new species (and others in this study) apart from other members of the *P.nuntia* complex. The gap in all three species described here is about ‘as wide as palpophore’, which is similar to *P.nuntia* according to Villalobos-Guerrero (2019), but differs from the Southern Ocean species *P.latipalpa* (Schmarda, 1861) from South Africa and *P.vallata* from Chile in which the gap is only as wide as the palpostyle (Wilson and Glasby 1993; Villalobos-Guerrero 2019).

## ﻿Discussion

The present study supports the finding of Bakken and Wilson (2005) of the non-monophyly of *Perinereis* and the ‘*P.nuntia*’ species complex. Bakken and Wilson (2005) found that the clade containing the type species, *P.novaehollandiae* Schmarda, 1861 (a junior synonym *P.amblyodonta* Schmarda, 1861) does not group with the clade *P.nuntia+P.vallata*, suggesting that the characteristic arc of bars on area VI, may not be homologous between the two groups. Nevertheless, the presence of a large number (> 10) of uniform, very short bars in area VI may be found as an autapomorphy for some subgroups within the species group, for example, in the sister grouping of *P.damietta* and the Southern Ocean species, *P.vallata*. Possibly, fine details of paragnath form and pattern may be found to delineate natural groups within *Perinereis*, which would lend support to Villalobos-Guerrero’s (2019) recognition of the taxonomic importance of faint ridges and furrows of the dorsal oral ring. Microstructures of the pharyngeal surface probably reflect underlying muscular and therefore may play a role in the form and function of paragnaths.

Despite recent advancements in integrative studies in many groups of polychaetes, taxonomic confusion still exists in many groups of Nereididae. *Perinereis* species are especially problematic due to difficult morphological species differentiation and a lack of detailed systematic studies. This has led to informal denomination of the species complex and recognition of geographic morphs and varieties such as *P.cultrifera* (Scaps et al. 2000) and the *P.nuntia* species group (Wilson and Glasby 1993; Glasby and Hsieh 2006; Sampértegui et al. 2013). Today, genetic assessment in combination with morphology is considered an effective tool for redescription of several species principally focused on population differentiation (Rouabah and Scaps 2003) and species delimitation (Chen et al. 2002; Park and Kim 2007; Sampértegui et al. 2013; Villalobos-Guerrero et al. 2021). The present study confirms the utility of such an approach, and moreover demonstrates that the specific combination of the barcoding gene and selected morphometric characters is an effective way to delineate cryptic species.

Finally, this study has uncovered further examples of sympatry among polychaetes. All three new species described here were found in the same habitat, viz., intertidal sand and mud, under stones, at the same location. *Perinereisdamietta* appears to have a slightly wider habitat preference as it also occurs sub-tidally, but more intense sampling including exploration of potential microhabitat differences, is required to confirm our observations. Assuming sympatry, identification of the specific isolation mechanism(s) would be interesting. Several studies have suggested the importance of reproductive isolation as an important speciation mechanism in the species group (e.g., Yoshida 1984; Hardege and Bartels-Hardege 1995). This idea merits further investigation as an explanation for the phenotypic similarity of the three cohabiting nereidid species described in this study.

## Supplementary Material

XML Treatment for Perinereis nuntia

XML Treatment for Perinereis suezensis

XML Treatment for Perinereis fayedensis

XML Treatment for Perinereis damietta

## Supplementary materials

Supplementary material 1 Supplementary data Data type excel file. Explanation note Morphometric characterization of 45 specimens of three putative species of *Perinereis*, identified by voucher number and sample identification (as for Table 1). Abbreviations: p, present; NP, not present. https://binary.pensoft.net/file/775679This dataset is made available under the Open Database License (http://opendatacommons.org/licenses/odbl/1.0/). The Open Database License (ODbL) is a license agreement intended to allow users to freely share, modify, and use this Dataset while maintaining this same freedom for others, provided that the original source and author(s) are credited.Asmaa Haris Elgetany, Torsten H. Struck, Christopher J. Glasby
